# Climbing-Specific Exercise Tests: Energy System Contributions and Relationships With Sport Performance

**DOI:** 10.3389/fphys.2021.787902

**Published:** 2022-01-24

**Authors:** Marcin Maciejczyk, Michail Lubomirov Michailov, Magdalena Wiecek, Jadwiga Szymura, Robert Rokowski, Zbigniew Szygula, Ralph Beneke

**Affiliations:** ^1^Department of Physiology and Biochemistry, Faculty of Physical Education and Sport, University of Physical Education, Kraków, Poland; ^2^Department Theory and Methodology of Sports Training, National Sports Academy, Sofia, Bulgaria; ^3^Department of Clinical Rehabilitation, Faculty of Motor Rehabilitation, University of Physical Education, Kraków, Poland; ^4^Department of Alpinism and Tourism, Faculty of Tourism and Recreation, University of Physical Education, Kraków, Poland; ^5^Department of Sports Medicine and Human Nutrition, Faculty of Physical Education and Sport, University of Physical Education, Kraków, Poland; ^6^Institute of Sport Science and Motology, Philipps University, Marburg, Germany

**Keywords:** climbing, aerobic, anaerobic, physical fitness, performance, exercise testing

## Abstract

**Purpose:**

The aim of the study was to evaluate distinct performance indicators and energy system contributions in 3 different, new sport-specific finger flexor muscle exercise tests.

**Methods:**

The tests included the maximal strength test, the all-out test (30 s) as well as the continuous and intermittent muscle endurance test at an intensity equaling 60% of maximal force, which were performed until target force could not be maintained. Gas exchange and blood lactate were measured in 13 experienced climbers during, as well as pre and post the test. The energy contribution (anaerobic alactic, anaerobic lactic, and aerobic) was determined for each test.

**Results:**

The contribution of aerobic metabolism was highest during the intermittent test (59.9 ± 12.0%). During continuous exercise, this was 28.1 ± 15.6%, and in the all-out test, this was 19.4 ± 8.1%. The contribution of anaerobic alactic energy was 27.2 ± 10.0% (intermittent), 54.2 ± 18.3% (continuous), and 62.4 ± 11.3% (all-out), while anaerobic lactic contribution equaled 12.9 ± 6.4, 17.7 ± 8.9, and 18.2 ± 9.9%, respectively.

**Conclusion:**

The combined analysis of performance predictors and metabolic profiles of the climbing test battery indicated that not only maximal grip force, but also all-out isometric contractions are equally decisive physical performance indices of climbing performance. Maximal grip force reflects maximal anaerobic power, while all-out average force and force time integral of constant isometric contraction at 60% of maximal force are functional measures of anaerobic capacity. Aerobic energy demand for the intermittent exercise is dominated aerobic re-phosphorylation of high-energy phosphates. The force-time integral from the intermittent test was not decisive for climbing performance.

## Introduction

Sport climbing was included in the 2020 Olympic program due to the great increase in its popularity. In addition, the highest climbing achievements performed on rocks and not during competitions have increased asymptotically in the last three decades (Michailov, 2014). Both facts indicate that climbing has reached an advanced stage of development. This places higher demands upon climbers’ preparation and requires monitoring as well as evaluating climbing-specific fitness to optimize training and further increase climbing performance. It has been shown that traditionally used exercise tests are not useful in the assessment of climbers’ training state (Watts, 2004). In order to select appropriate exercise tests for climbers, one should be well-acquainted with specific load characteristics, performance limiting factors, and physiological aspects in climbing.

There are many climbing disciplines, differing in duration and exercise intensity. During competitions, the time limit of lead climbing is 6 min. Otherwise, the ascents on sport climbing routes (leading) are usually 1–4 min (red-point—after working out the route) and 3–10 min long (on-sight—first attempt). Bouldering ascents usually last 30–50 s (Michailov, 2014). During a bouldering competition, the climbers may attempt a boulder problem as often as they want, and can do so in 4 or 5 min. After that, they rest for 4 or 5 min and then start working on the next boulder problem. The actual 15-m speed climbing men’s record is 5.21 s. Consequently, climbing is not equivalent to permanent maximal effort but it is a mix of distinct patterns of muscular efforts determined by contraction intensity related to maximal force, duration of contraction phases, and their relation to relaxation phases. Typical for all climbing disciplines is that they demand strenuous intermittent isometric muscle contractions (Sheel, 2004). The contraction time of the finger flexor muscles is much longer than their relaxation time. The contraction to relaxation ratio is blood-flow restricting. It can be 4:1 in sport climbing and 13:1 in bouldering (Schadle-Schardt, 1998; White and Olsen, 2010).

The structure of climbing performance comprises a large set of motor abilities and skills, including physiological and psychological factors, anthropometric characteristics, and flexibility (Sheel, 2004; Watts, 2004; Giles et al., 2006; Michailov, 2014). The physical variables, which largely explain the variance in climbing performance, are trainable factors such as finger-arm strength and endurance, whereas anthropometric characteristics and flexibility have comparably small effects (Mermier et al., 2000; Baláš et al., 2012; Laffaye et al., 2016). Physical, technical, and mental characteristics explain the structure of climbing performance in a similar way, which may serve as evidence that climbers need to conduct harmonious development training (Magiera et al., 2013).

From a physiological point of view, climbing is an interesting discipline because it requires: (a) a satisfactory level of aerobic power and general endurance, and (b) specific muscular strength and endurance supplied by aerobic, phosphagen [adenosine triphosphate (ATP) and phosphocreatine (PCr)], and anaerobic lactic energy systems (Sheel, 2004; Watts, 2004; Giles et al., 2006; Bertuzzi et al., 2007). Previous studies were focused on the physiological response during climbing (Watts and Drobish, 1998), finger flexor strength, and endurance (MacLeod et al., 2007; Baláš et al., 2012; Philippe et al., 2012), as well as aerobic power (Billat et al., 1995). During climbing, cardiopulmonary measures of approximately 75% maximal oxygen consumption (VO_2_max) and 83% maximal heart rate or lactate concentrations (Lac) exceeding 4.5 mmol⋅L^–1^ suggest substantial contribution of both, aerobic and anaerobic glycolytic energy (Booth et al., 1999; Giles et al., 2006). Moreover, highly trained climbers can perform repetitive isometric contractions of the forearm without fatigue, whilst tolerating high levels of acidosis indicating high anaerobic power, buffer capacity, and lactate removal (Giles et al., 2006; Michailov et al., 2015, 2017). Although repetitive isometric contractions in climbing are of anaerobic nature, ATP-PCr recovery and lactate removal require efficient aerobic metabolism. Thus, oxygen consumption remains elevated into the post-climb recovery period (Watts, 2004). Billat et al. (1995) noted that during climbing, heart rate is high for a relatively low oxygen uptake level. They concluded that oxidative metabolism may play a secondary role in rock climbing. Similarly, Sheel et al. (2003) observed a disproportional rise in heart rate compared to oxygen uptake during climbing. However, they concluded that climbing requires not only anaerobic but also aerobic metabolism. Only Bertuzzi et al. (2007) evaluated the energy contributions during real climbing and indicated that climbing predominantly involves the aerobic and anaerobic alactic systems.

Sport-specific exercise testing is a difficult task in climbing. There is a lack of research equipment specific to this discipline, and for that reason, standard biomechanical measurements (handgrip dynamometers) or physiological measurements were performed *via* VO_2_max measurements on a treadmill/cycle ergometer or by comparing physiological responses during real climbing to the results of these standard tests (Sheel et al., 2003). However, traditional ergometer tests reflect general fitness level. They are not specific to climbing. Corresponding test results did not correlate with climbing performance (Michailov et al., 2015). Unlike maximal treadmill or cycle ergometer tests, the pattern of physiological responses during climbing tests do not allow to determine submaximal performance markers comparable to lactate or ventilatory thresholds. Therefore, the interpretation of data obtained in these tests is limited when used to establish relative intensities for training through climbing (Watts, 2004; Schöffl et al., 2006). This is most likely due to isometric muscle contractions and holding one’s breath during climbing, as well as the fact that climbing test results depend on muscle strength, aerobic, and anaerobic metabolism (Watts et al., 2000; Michailov et al., 2017).

Exercise tests for climbers should reflect load characteristics, sport technique, and fatigue in climbing. However, testing should not completely mimic actual climbing because the intensity and duration should be assigned according to the ability the tests are intended to assess. Therefore, many researchers have focused on testing climbers’ finger strength and endurance (MacLeod et al., 2007; Philippe et al., 2012; Baláš et al., 2016; Michailov et al., 2018; Fryer et al., 2021) using different test protocols and climbing-specific dynamometers, while expressing the intensity as a percentage of maximal voluntary contraction (MVC). These types of assessments distinguished climbing groups of different ability levels and appear more informative because they are likely to induce similar patterns of muscle fiber-type activation, metabolism, and fatigue compared to real climbing situations. Moreover, some authors have used a combination of different muscle endurance tests (continuous, intermittent, and all-out) in an attempt to assess climbers’ aerobic or anaerobic capacity at peripheral levels (Baláš et al., 2016; Michailov et al., 2018). Most of the parameters for these endurance tests were highly reliable (ICC between 0.845 and 0.921) and valid for climbing performance (Michailov et al., 2018). Nonetheless, no energy system contribution has been assessed so far (anaerobic alactic, anaerobic lactic, and aerobic) during a laboratory test and using a validated device dedicated to climbers’ physical fitness examination. Determination of the energy contribution during climbing-specific finger muscle endurance tests shall allow to identify how informative these tests are, with respect to local aerobic or anaerobic capacity assessment and relative energy contribution, compared to real climbing.

Therefore, the aim of this study was to evaluate distinct performance indicators and energy system contributions in 4 different finger flexor muscle exercise tests, performed using an apparatus developed for comprehensive assessment of physical fitness in climbers.

## Materials and Methods

### Study Design

The test battery included (a) a maximal finger strength test followed by 3 different finger flexor muscle endurance tests, (b) an all-out test (30 s), (c) a continuous, and (d) an intermittent endurance test. Tests (b) to (d) were conducted in random order. The maximal finger strength assessment served to set the relative intensity of tests (c) and (d). Before, during, and after the muscle endurance tests, gas exchange data were collected. The tests were performed with the climber’s preferred hand. Blood was drawn from the fingers of the opposite hand. Pre- and post-test blood lactate levels were measured. Energy contribution (anaerobic alactic, anaerobic lactic, and aerobic) was determined for each muscle endurance test. All tests were performed on 1 day at temperatures between 20 and 22°C, and the interval between tests was approximately 1 h. Participants were asked to avoid intense physical efforts in the 2 days before testing.

### Participants

Thirteen healthy, experienced male climbers volunteered for this study. Their current climbing ability level ranged from intermediate to higher elite according to the grading comparative table of the International Rock Climbing Research Association (IRCRA) (Draper et al., 2015). The participants’ current, mean climbing grade (French grading system) in the red-point style (highest difficulty of a route, which a climber can climb after the route has been previously rehearsed) was 8a + (range 7a–9a French/sport grade). To enable statistical analysis, these grades were converted using the metric IRCA scale (Draper et al., 2015) and then presented along with other detailed characteristics of the participants in Table 1. The criteria for inclusion in the study were: practicing sport (lead) climbing, possessing a minimum current climbing level of 7a (French/sport grade), being an active climber performing at least two climbing specific-training sessions per week, and no injury in the 6 months preceding the study. Seven of our participants combined sport climbing and bouldering, although they were training to manage harder outdoor sport climbing routes. The other 6 participants regularly practiced sport climbing and specialized in alpine ascents.

**TABLE 1:** Participants’ characteristics.

Variables	Mean ± SD
Age	29.4 ± 7.88
BH (cm)	178.2 ± 4.9
BM (kg)	69.5 ± 7.6
BMI	21.8 ± 1.8
FAT (%)	12.6 ± 2.9
FM (kg)	8.8 ± 2.2
LBM (kg)	60.7 ± 7.1
AS (cm)	184.6 ± 5.9
**Circumferences (cm)**	
Arm	27.6 ± 3.2
Thigh	47.2 ± 7.0
Calf	33.4 ± 3.6
Climbing experience (years)	14.54 ± 5.50
Current red-point (IRCRA scale)	23.69 ± 3.92
Current on-sight (IRCRA scale)	20.85 ± 3.13
Current boulder grade (IRCRA scale)	23.56 ± 2.19

*BH, body height; BM, body mass; BMI, body mass index; FM, fat mass; LBM, lean body mass; AS, arm span.*

### Anthropometry

The body height was measured to the nearest 0.1 cm with a stadiometer (Seca, Germany). Body mass and composition (method of bioelectrical impedance) were determined using the Jawon scale (Korea). Arm span was measured in a standing position with the arms abducted horizontally at the height of the shoulders. Arm, thigh, and calf circumferences were measured at the site of the largest circumference and with the muscles relaxed.

### Apparatus, Testing Position, and Warm-Up

Finger strength and endurance tests were performed on the 3DSAC, which is an advanced climbing-specific apparatus developed for comprehensive performance evaluation in climbers, as described in detail by Michailov et al. (2018). 3DSAC is composed of: (a) a 3D force measuring module (measuring range ± 2 kN, comprehensive accuracy 0.5%, 12 bit accuracy of the analog-to-digital converter, 125 Hz sample rate), calibrated for a wooden 23-mm deep climbing hold; (b) real-time feedback guidance module enabling the participants to control the intensity and duration of muscle contractions and the rest intervals; (c) construction for adjusting the position of the climbing hold according to the participants’ height and arm length; and (d) a software package allowing to create different test protocols and to precisely calculate mechanical parameters.

**FIGURE 1:**
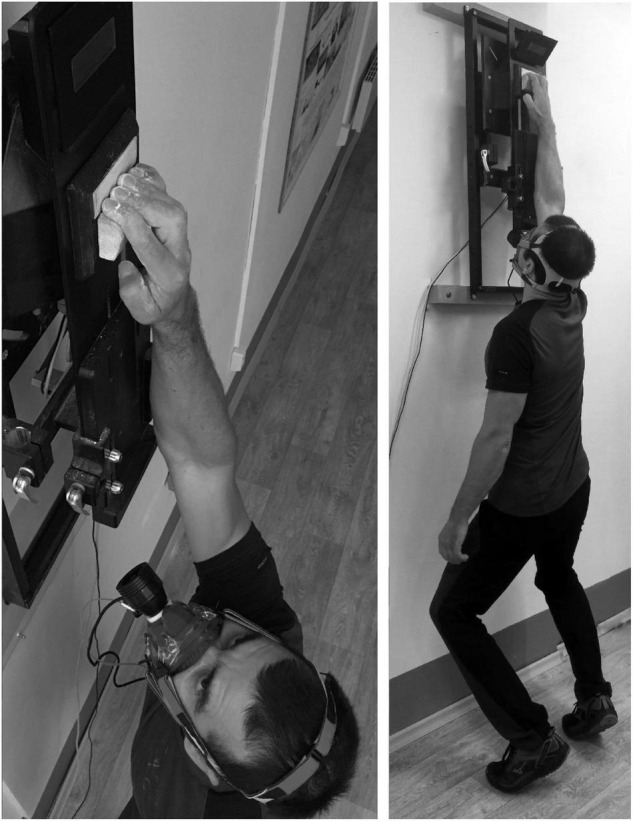
Climber’s position during exercise test.

A familiarization session was held before the study began. The participants were instructed on how to perform the tests (goal of the test and related actions, body position, and grip) and then familiarized with the device as well as technique of performing the tests. During the familiarization session, body position was corrected by the test supervisors. After confirming proper test technique, the climber was allowed to perform the test. During the warm-up and all of the tests, the participants stood facing the 3DSAC, and they used an open finger grip position on the 23-mm hold, which was mounted on the force measuring module (Michailov et al., 2018). Their shoulders were flexed at a 180-degree angle, with their elbows fully extended. Maintaining the grip, the participants flexed their knees to load the hold with their body weight (Figure 1). During testing, the feet were on the floor. In order not to allow climbers with high levels of strength to hang with their feet off the ground, they wore a weight vest during the maximal strength and all-out tests.

The warm-up procedure was identical to the one used by Baláš et al. (2016) and Michailov et al. (2018): 5 min of stair walking and 2 sets of eight, 5-s muscle contractions on the 3DSAC, applying a force of 30% body mass, alternated by 5-s rest intervals.

### Maximal Strength Test

The maximal strength tests included 3 maximal voluntary finger flexor contractions separated by 1-min rest intervals. The maximal strength was determined as the highest force value from the 3 trials. The participants, for whom maximal force (F_max_) was more than their body mass, were wearing weight vests, which did not allow them to hang on the hold. F_max_ and F_max_ related to body mass (relF_max_) were registered during the maximal strength test. The first muscle endurance test was performed 10 min after the maximal strength test.

### All–Out, Continuous, and Intermittent Tests

In the all-out test, climbers had to quickly develop maximal force and maintain the maximal effort for 30 s. In the all-out test, peak force (F_peak_), average force (F_avg_), and fatigue index (FI) were used. FI was calculated *via* the following equation:


FI=(F-peakF)end-of-test÷F×peak100

During the continuous test, the participants had to develop a force corresponding to 60% of F_max_ and maintain the force in a target zone totaling ± 10% of the target force for as long as possible. In the intermittent test, the intensity and target zone were the same as in the continuous test. During the intermittent test, the participants alternated contraction and relaxation intervals of 8 and 2 s, respectively. The intermittent test was also performed until the climbers were not able to maintain force in the target zone. Both the continuous and intermittent tests were automatically stopped when the force dropped below the target zone for more than 1 s.

In the continuous and intermittent tests, time in the target zone (T_*tz*_), force-time integral (FTI), and FTI related to body mass were analyzed. In the intermittent test, the number of muscle contractions was also considered.

### Calculation of Aerobic, Lactic, and Alactic Energy Contribution

Before (5 min), during and after the exercise tests (10 min), oxygen uptake (VO_2_), and respiratory exchange ratio (RER) were measured continuously (breath-by-breath method) using an ergospirometer (Cortex MetaLyzer, Germany). The device was calibrated before each test according to the manufacturer guidelines.

The blood samples were collected by pricking the finger at rest and in the 1st, 2nd, and 3rd min post-test for the determination of lactate concentration. Plasma lactate concentration was measured *via* enzymatic colorimetry using the Lactate PAP (bioMerieux, France). Assay sensitivity amounted to 0.07 mmol⋅L^–1^. The assay was linear up to 10 mmol⋅L^–1^. The absorbance was measured at 505 nm using the Thermo Scientific Evolution 201 UV/VIS spectrophotometer (United States). The post-exercise increase in lactate concentration (ΔLac) was also calculated.

The energy contribution for each test was determined as previously described (Beneke et al., 2002, 2004; Bertuzzi et al., 2007; Artioli et al., 2012). In brief, aerobic contribution (net) (VO_2E*x*_) was calculated from oxygen uptake above rest during the exercise test and the energy equivalent of O_2_ being assumed from 19.6 to 21.1 kJ⋅L^–1^ (depending on RER). To estimate the anaerobic lactic contribution, the value of 1 mmol⋅L^–1^ ΔLac was considered equivalent to 3 mLO_2_⋅kg^–1^, e.g., 63 kJ⋅kg^–1^ (di Prampero and Ferretti, 1999). The contribution of the anaerobic alactic system was estimated using the fast component of excess post-exercise oxygen consumption (VO_2E*POC*_) (Beneke et al., 2004; Bertuzzi et al., 2007). Total energy contribution was calculated as the sum of the 3 energy systems. In addition, the contributions of these 3 systems were also expressed as percentages in relation to total energy contribution.

### Statistical Analysis

All calculations were carried out using Microsoft Excel and the IBM Statistical Package for Social Sciences (SPSS) for Windows (Version 22, Chicago, IL). Data are reported as means and SDs. Differences between the types of energy contribution from the muscle endurance tests were analyzed through ANOVA. The *post hoc* analysis was performed using Bonferroni’s test. Effect sizes were presented as partial eta squared (η*_*p*_*^2^). Pearson’s correlation coefficients were calculated to estimate correlations between the mechanical parameters and climbing ability. Statistical significance was set at *p* ≤ 0.05.

## Results

The average force in the all-out test was 404 ± 46 N and FI was 38.0 ± 14.4%. The time in the target zone for the intermittent test was 2.5-fold longer (*p* = 0.018) than in the continuous test. The relative force-time integral in the intermittent test was significantly (*p* = 0.049) higher than in the continuous test. There were significant correlations between climbing ability and relF_max_ in the maximal strength test (*r* = 0.812, *p* = 0.001), relF_avg_ in the all-out test (*r* = 0.816, *p* = 0.001), and relFTI in the continuous test (*r* = 0.719, *p* = 0.008) (Table 2).

**TABLE 2:** Results of exercise tests (mean ± SD for all climbers).

Variables	Result	Correlation with climbing
**Maximal strength test**		
F_max_ (N⋅kg^–1^)	8.57 ± 1.24	0.812 (*p* = 0.001)
**30 s all-out test**		
F_peak_ (N⋅kg^–1^)	7.73 ± 1.27	0.349 (*p* = 0.243)
F_avg_ (N⋅kg^–1^)	5.81 ± 0.66	0.816 (*p* = 0.001)
FI (%)	38.00 ± 14.42	−0.266 (*p* = 0.404)
**Intermittent test**		
# Reps	23.62 ± 17.93	0.230 (*p* = 0.449)
T_*tz*_ (s)	151.31 ± 111.5	0.230 (*p* = 0.449)
FTI (N.s⋅kg^–1^)	735.44 ± 542.76	0.407 (*p* = 0.167)
**Continuous test**		
T_*tz*_ (s)	60.05 ± 53.30	0.195 (*p* = 0.544)
FTI (N.s⋅kg^–1^)	301.90 ± 75.19	0.719 (*p* = 0.008)

*F_max_, maximal force; F_peak_, peak force; F_avg_, average force; FI, fatigue index; T_tz_, time in target zone; FTI, force-time integral; p-level of significance.*

The highest ΔLac was noted after the intermittent test (2.17 ± 1.14 mmol⋅L^–1^), which was greater (*p* = 0.002) than that observed following the continuous test (0.89 ± 0.6 mmol⋅L^–1^). After the continuous test, VO_2E*POC*_ was lower than that post the all-out test (*p* = 0.02) and that following the intermittent test (*p* = 0.049). VO_2E*x*_during exercise was similar (*p* > 0.05) for the all-out and the continuous tests. In the intermittent test, VO_2E*x*_ was considerably higher than during the all-out (*p* < 0.001) and continuous tests (*p* < 0.001) (Table 3).

**TABLE 3:** Total energy contribution and energy system contribution in particular tests (mean ± SD for all climbers).

Energy system contribution	All-out test	Intermittent test	Continuous test	*p*-value	(η*_*p*_*^2^)
**Total energy contribution**					
(J⋅kg^–1^)	499.4 ± 186.8	1057.6 ± 436.6	314.8 ± 108.6	0.002	0.655
**Anaerobic lactic**					
(J⋅kg^–1^)	90.6 ± 45,9	136.4 ± 71.9	55.7 ± 37.5	0.007	0.397
ΔLac (mmol⋅L^–1^)	1.44 ± 0.73	2.17 ± 1.14	0.89 ± 0.6	0.007	0.399
**Anaerobic alactic**					
(J⋅kg^–1^)	311.7 ± 131.8	287.7 ± 95.1	170.6 ± 109.5	0.007	0.466
VO_2E*POC*_ (mL⋅kg^–1^)	15.14 ± 6.37	14.02 ± 4.66	8.37 ± 5.53	0.007	0.462
**Aerobic**					
(J⋅kg^–1^)	97.1 ± 55.4	633.5 ± 390.3	88.5 ± 43.9	*p* < 0.001	0.657
VO_2E*x*_ (mL⋅kg^–1^)	4.80 ± 2.81	30.89 ± 18.51	4.33 ± 2.18	*p* < 0.001	0.669

*ΔLac–difference between peak blood lactate and lactate at rest, VO_2EPOC_–oxygen uptake (net) during fast component of excess post-exercise oxygen consumption, VO_2Ex_-oxygen uptake (net) during exercise.*

**FIGURE 2:**
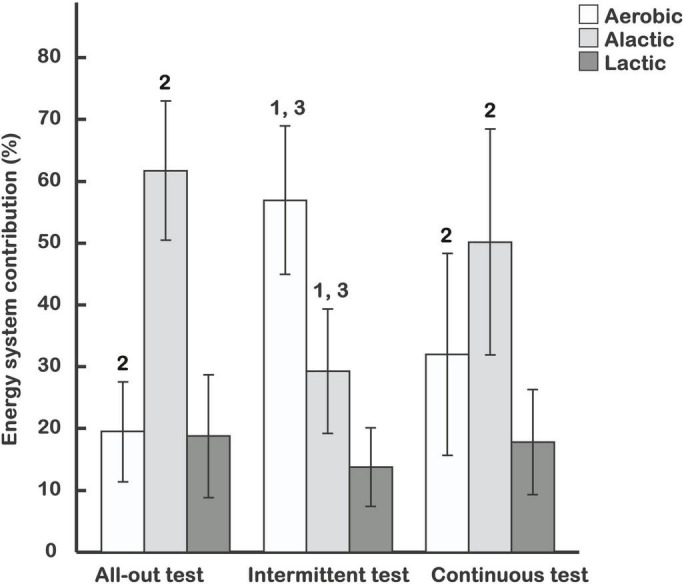
Aerobic, alactic, and lactic relative energy contributions (mean ± SD) in 3 exercise tests proposed for climbers. 2—when significantly different (*p* < 0.05) from the intermittent test; 1, 3—when significantly different (*p* < 0.05) from the all-out and continuous test, respectively.

The total energy requirement for the intermittent test was approximately 3.4 times that demanded for the continuous (*p* = 0.002), and twice as high as for the all-out test (*p* = 0.033), which required more energy than the continuous test (*p* = 0.003) (Table 3). The contribution of aerobic metabolism was 59.9 ± 12.0% in the intermittent test, 28.1 ± 15.6% in continuous exercise, and 19.4 ± 8.1% in the all-out test. The corresponding contributions of anaerobic alactic energy were 27.2 ± 10.0, 54.2 ± 18.3, and 62.4 ± 11.3% (all-out test), while relative anaerobic lactic energy was 12.9 ± 6.4, 17.7 ± 8.9, and 18.2 ± 9.9%, respectively (Figure 2). There were no significant differences (*p* = 0.296) between the tests in relative anaerobic lactic contributions. However, the relative aerobic energy contribution of the intermittent test was higher than in the continuous (*p* = 0.004) and all-out test (*p* < 0.001). The continuous and all-out tests did not differ with respect to aerobic energy (*p* = 0.108). The relative alactic energy contribution in the intermittent test was significantly smaller than in the continuous (*p* = 0.006) and all-out (*p* < 0.001) tests. The alactic energy contribution in the continuous and all-out tests did not differ (*p* = 0.136).

## Discussion

In rock climbing, the gravity force is countered by grip fixation and active hanging position using the upper limbs as a fix point comparable to a relative calm pendulum or with leg-support, ideally comparable to a horizontally attached uneven tripod. Consequently, climbing specific-grip performance imposes a trainable physical factor, mostly contributing to variance of climbing performance (Mermier et al., 2000; Baláš et al., 2012; Laffaye et al., 2016).

For the first time, a test battery combining 4 distinct tests was designed to identify grip performance indicators in terms of a standardized diagnostic tool. This was achieved by measuring F_max_ *via* the MVC test and the relating the 30-s all-out grip performance, 60% of F_max_ continuous performance until failure to sustain the 60% target, and 60% of F_max_ intermittent performance until failure to repeat the 60% target to F_max_. This test battery combines objectively determined performance indicators, clearly defined by endpoints of a duration set as 30 s all-out or sustainability of a given level of performance within set limits of tolerance. Additionally, the test battery should mimic the pattern of overcoming gravity forces in rock climbing with an analysis of corresponding aspects of metabolic demands in elite climbers.

The present approach extends the previously described high correlation between F_max_ and climbing ability, identifying comparable explanations of variance for climbing performance, further explained by F_avg_ in the 30-s all-out test and force-time integral in the continuous 60% F_max_ test. This strong interrelationship was neither seen in net contraction time (during both continuous and intermittent tests) nor in the overall duration or force-time integral of the intermittent test. At first glance, the latter finding appears surprising, as the intermittent 60% F_max_ test also measures the sustainability of a given F_max_ fraction. The intended contraction (8 s) to relaxation (2 s) ratio of 4:1 increased the sustainability regarding the 60% of F_max_ intermittent performance test to the overall duration (total contraction plus relaxation time) of ∼234 s. The real net contraction time (the time when the force was applied below the target zone added to the time when the force was within the target zone) detected *via* the force time curve of every single contraction was 171 s shifting the contraction (7.3 s) to relaxation (2.7 s) ratio toward relaxation by ∼32%. The overall duration of 234 s reflects an extremely long-lasting red-point or very short on-sight, first-try sport climbing conditions. In hindsight, this test-specific pattern of intensity and duration appeared potentially suboptimal with respect to the tested climbers. Six of them were alpinists who primarily focused on practicing the on-sight type, with their first attempt in sport climbing usually lasting longer than 4 min. The remaining 7 participants combined bouldering with sport climbing lasting between 30 and 50 s and longer than 4 min, respectively. Thus, there is a fair possibility that the settings of the intermittent test resulted in a sustainability profile that was rather unspecific with respect to the specialization of the tested athletes. The great variability in the intermittent test performance may serve as a support for the idea of limited specification of the 60% intermittent test, with special respect to performance limits of the climbing events preferred by the tested athletes.

The average force of the all-out test was ∼75% F_peak_, combined with a FI of ∼40%. This fits well into the pattern of performance and fatigue seen in the Wingate Anaerobic Test (WAnT) if the participant remains seated on the ergometer saddle throughout the test (Inbar et al., 1996). Remaining seated throughout the WAnT, active stabilization of the body on the ergometer during test using the arm and trunk muscles as counterbalance and the requirement for an effective transfer of leg-power onto the pedals, makes the WAnT a highly anaerobic whole-body exercise. The relative anaerobic metabolic cost of the WAnT reflects ∼80% of the total metabolic energy shared as ∼50% anaerobic glycolytic and ∼30% anaerobic alactic energy (Beneke et al., 2002). In a typical WAnT, failure to produce 60% of peak power comes along with ΔLac in the size of ∼14 mmol⋅L^–1^. Under the assumption that the distribution space of lactate reflects approximately 45% of the body mass (di Prampero, 1981; Mader and Heck, 1986; Beneke, 2003) and muscle mass was ∼40% of the body mass, the latter lactate response is equivalent to an increase of ∼10.5 mmol⋅kg^–1^ body mass or 26 mmol⋅kg^–1^ wet muscle in 30 s. The increase in muscle lactate substantially limits glycolytic rate, as demonstrated in all-out cycling tests lasting from 15 to 60 s. The corresponding decrease in PCr was ∼20 mmol⋅kg^–1^ wet muscle, combined with an average net VO_2_ from ∼35 to 40 mL⋅kg^–1^ wet muscle over 30 s (Beneke et al., 2002, 2005, 2007; Wittekind et al., 2011, 2012; Leithäuser et al., 2015).

**FIGURE 3:**
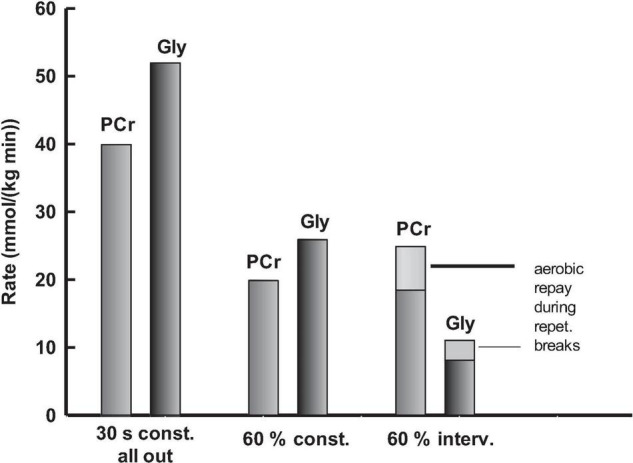
Anaerobic [phosphocreatine (PCr) and glycolytic (Gly)] system contributions of dominantly stressed muscles in exercise tests.

Also, the present 30-s all-out test has an overall relative anaerobic energy demand of ∼80% (Figure 2). However, with ∼18% anaerobic glycolytic and ∼62% anaerobic alactic energy (Table 2), the distribution between sources of anaerobic energy is pretty much the opposite of that seen in the WAnT. In highly trained athletes, isometric contractions of ∼50% F_max_ led to complete vascular occlusion (Barnes, 1980). Therefore, the above differences, between energetics of WAnT and present in the 30-s all-out test, likely reflect the substantially smaller mass of the muscle dominantly stressed during isometric force development and vascular occlusion in the single-handed hanging task, as supported by our metabolic results. With reference to the above-assumptions concerning lactate water space and muscle mass, ΔLac of 1.44 mmol⋅L^–1^ measured as the maximum blood lactate increases shortly after test termination, and reflects 1.1 mmol⋅kg^–1^ body mass or 2.8 mmol⋅kg^–1^ muscle mass in 30 s. If this net lactate level results mainly from an increased glycolytic rate in performance limiting muscles with corresponding limited glycolytic rate due to a ∼26 mmol⋅kg^–1^ increase in muscle lactate, then, ∼11% of the total muscle mass appears to be the dominantly stressed muscle fiber mass in this specific test. The anaerobic alactic energy equals a decrease in PCr of ∼4.7 mmol⋅kg^–1^ body mass or ∼11.8 mmol⋅kg^–1^ total muscle mass. Furthermore, ∼2.2 mmol⋅kg^–1^total muscle mass would reflect a performance limiting decrease of ∼20 mmol⋅kg^–1^ if the dominantly stressed muscle is ∼11% of the total muscle mass. This is well within the range of limits for the dynamics of cellular ATP breakdown and re-phosphorylation rates of mixed skeletal muscle at maximum isometric contraction measured using magnetic resonance spectroscopy or histochemical methods (Francescato et al., 2008; Barclay, 2017). The corresponding decrease in 89% of the assisting tissue was ∼10.7 mmol⋅kg^–1^ during 30 s (Figure 3). The net VO_2_ was lower than in tests with high frequent contraction and relaxation cycles. Due to vascular occlusion in the isometrically stressed dominant muscles, the net VO_2_ of 4.8 mL⋅kg^–1^ body mass is likely attributed to the aerobic metabolism of the assisting tissue, equivalent to ∼13.5 mL⋅kg^–1^ assisting muscle mass.

A FI of ∼40% in the 30-s all-out test and failure to produce 60% of F_max_ at test termination of the two 60% tests, respectively, suggest similar levels of fatigue concerning the performance-limiting muscle mass. The 60% F_max_ continuous test lasted ∼60 s. It generated a ΔLac of 0.89 mmol⋅L^–1^ quickly released from the dominantly stressed muscles after test termination *via* maximized reperfusion and quick distribution to blood and body water. This is equivalent to 0.7 mmol⋅kg^–1^ body mass or 1.7 mmol⋅kg^–1^ muscle mass. Under the assumption that this reflects a ∼26 mmol⋅kg^–1^ performance-limiting muscle lactate level, the dominantly stressed fiber volume totals ∼7% of the total muscle mass. This is ∼60% of the all-out work value, further supported by Henneman’s size principle (Henneman, 1957). The anaerobic alactic energy of the constant 60% F_max_ test equaled a decrease in PCr of 2.6 mmol⋅kg^–1^ body mass or 6.4 mmol⋅kg^–1^ total muscle mass. Consequently, ∼1.4 mmol⋅kg^–1^ total muscle mass can be attributed to a performance limiting PCr decrease of ∼20 mmol⋅kg^–1^ dominantly stressed 7% muscle mass. This leaves a moderate decrease of ∼5.4 mmol⋅kg^–1^ in the remaining 93% assisting muscle mass (Figure 3). The latter goes hand in hand with a net VO_2_ of ∼11.6 mL⋅kg^–1^ assisting muscle. Thus, the PCr and VO_2_ seem to indicate that the 60% intensity reduced the strain on assisting muscles by almost 60% compared to the all-out condition, resulting in shares of ∼18% glycolytic, ∼54% anaerobic alactic, and ∼28% aerobic energy, which appear slightly more anaerobic than the results obtained for tests of comparable duration and implementing large muscle masses (Figure 2).

**FIGURE 4:**
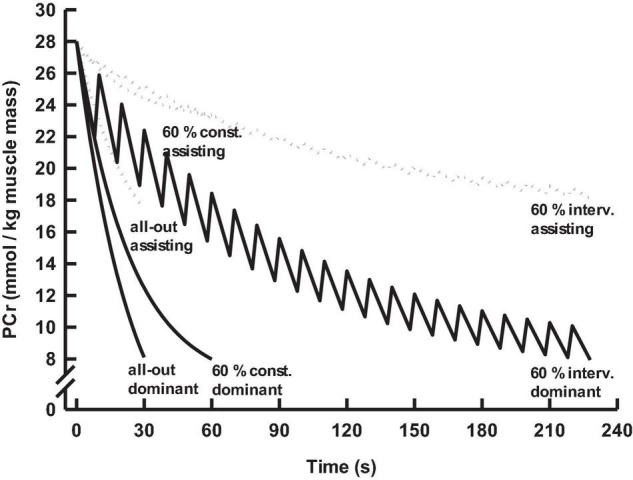
Phosphocreatine decrease in the dominantly stressed and assisting muscles during the muscle endurance tests.

A contraction to relaxation ratio of 2.7 increased the sustainability of the 60% F_max_ intermittent performance test to an overall duration of ∼234 s, with a ∼171 s net contraction time. Alternation of contraction and relaxation enables intermittent perfusion of the dominantly stressed muscles, oxygen supply, aerobic pyruvate/lactate utilization, and re-phosphorylation of high-energy phosphates. The overall net VO_2_ was ∼30.9 mL⋅kg^–1^ body mass in ∼234 s. The 60% of F_max_ continuous performance test generated a net VO_2_ of ∼11.6 mL⋅kg^–1^ assisting muscle, which equals ∼33.1 mL⋅kg^–1^ of the assisting muscle or 12.3 mL⋅kg^–1^ of body mass during the contraction periods of the intermittent test. The remaining 18.6 mL⋅kg^–1^ of body mass is used during the recovery intervals. Moreover, 1 mL of oxygen utilizes 0.015 mmol of pyruvate/lactate. This equals a pyruvate/lactate consumption of ∼0.28 mmol⋅kg^–1^ body mass during the recovery phases throughout the test. Aerobic pyruvate/lactate utilization is regulated by pyruvate dehydrogenase (PDH). Activators of the PDH are pyruvate, CoA, and NAD^+^, allosteric cofactors including Mg^2+^, Ca^2+^, and Mn^2+^ (Spriet and Heigenhauser, 2002; Strumilo, 2005). Consequently, the aerobic pyruvate/lactate consumption can be seen as a re-phosphorylation source of high-energy phosphates in the intermittently re-perfused dominantly stressed muscles. Additional net lactate release accumulated to 2.17 mmol⋅L^–1^ or ∼1.6 mmol⋅kg^–1^ of body mass, increasing the time until a critically performance limiting intramuscular lactate concentration had been reached in the dominantly stressed muscle. The fast component of post-test VO_2_ corresponds to ∼4.3 mmol⋅kg^–1^ body mass or ∼10.8 mmol⋅kg^–1^ muscle mass, of which 1.4 mmol⋅kg^–1^ do reflect the decrease of ∼20 mmol⋅kg^–1^ in the 7% dominantly stressed muscles and the remaining 9.4 mmol⋅kg^–1^ of muscle mass equaled a decrease in high-energy phosphates of ∼10.1 mmol⋅kg^–1^ assisting tissue at test termination (Figure 4). These decreases in high-energy phosphates came on top of ∼14.6 mmol⋅kg^–1^ muscle mass PCr-repay generated *via* aerobic use of 18.6 mmol⋅kg^–1^ body mass oxygen and ∼0.28 mmol⋅kg^–1^ body mass pyruvate/lactate during repetitive recovery intervals throughout the test. The 14.6 mmol⋅kg^–1^ total muscle mass PCr-repay was split according to the dominantly stressed and assisting muscles. During the initial 7.3 s of isometric contraction at 60% F_max_, the estimated dynamic decrease in PCr was ∼20% of the baseline level (Francescato et al., 2008; Barclay, 2017), and partly re-phosphorylated during the short relaxation periods without vascular occlusion (Figure 4). In the dominantly stressed 7% of muscle mass, this demand accumulated to ∼73 mmol⋅kg^–1^, combined with a PCr -payback of ∼53 mmol⋅kg^–1^ muscle, which leads to a PCr decrease of ∼20 mmol⋅kg^–1^ muscle mass equivalent to ∼0.6 mmol⋅kg^–1^ of body mass. The accumulated PCr demand totaling 93% of assisting muscle mass was ∼23 mmol⋅kg^–1^ muscle, partly compensated by a PCr payback of ∼12 mmol⋅kg^–1^, which further led to a decrease in PCr equaling ∼10 mmol⋅kg^–1^ of assisting muscle mass or ∼3.7 mmol⋅kg^–1^ body mass, leaving ∼4.3 mmol⋅kg^–1^ body mass for post-test PCr replenishment.

Considering aerobic pyruvate/lactate utilization and aerobic re-phosporylation during the relaxation phases of the intermittent test provides a modified image as compared with the shares of ∼13% glycolytic, ∼27% anaerobic alactic, and ∼60% aerobic energy as shown in Figure 2. The net lactate utilization of 0.28 mmol⋅kg^–1^ of body mass during subsequent recovery phases of the intermittent test recovery led to an underestimation of the glycolytic energy during accumulated contraction phases by ∼18 J or ∼13%, resulting in an overall underestimation of the total energy demand by ∼2%. Aerobic utilization of 0.28 mmol⋅kg^–1^ body mass lactate requires an oxygen uptake of ∼18.7 mL⋅kg^–1^, equivalent to ∼390 J⋅kg^–1^, which is in the same size as ∼99 J⋅kg^–1^ of body mass for an accumulated recovery of ∼53 mmol⋅kg^–1^ PCr of the dominantly stressed muscles, plus ∼300 J⋅kg^–1^ used for ∼12 mmol⋅kg^–1^ PCr payback of the assisting muscles. The latter process does not affect the overall shares of aerobic and anaerobic alactic energy. However, it allows to indicate that ∼63% of aerobic energy serves the recovery of high-energy phosphates during repetitive relaxation phases. If this fraction offers additional diagnostic value within the context of combined constant and standardized intermittent loads, then performance testing remains open for future investigation.

The combined analysis of performance and metabolic features of the present test battery, including the measurement of F_max_ *via* the MVC test and the relating 30-s all-out grip performance, 60% of F_max_ continuous performance until failure to sustain the 60% target, and 60% of F_max_ intermittent performance until failure to repeat the 60% target to F_max_, extended the well-known interrelationship between climbing performance and F_max_ through similar correlations between climbing performance and the 30-s all-out isometric F_avg_ and isometric FTI at 60% F_max_. Therefore, not only maximized grip force as a performance determinant in climbing but also all-out isometric contractions of a given duration or the ability to sustain a given percentage of F_max_ are equally decisive physical performance indicators. Although similar with respect to explaining the variance of climbing performance, the latter 3 tests evaluate different physiological performance limits. F_max_ allows to identify the ability of synchronized activation of relevant motor units, and thus, metabolic rate in terms of maximal anaerobic power. The observation of no interrelation between climbing performance and F_peak_ for the 30-s all-out test, which was lower than F_max_, seems to indicate teleoanticipation and therefore, a psycho-physiological limitation of maximized activation and synchronization of motor units, as well as utilization of maximum anaerobic power at this test modality. This limitation comes with the benefit of evidence that not only F_max_ or maximal anaerobic power, but also anaerobic capacity determine climbing performance. This was indicated by the strong correlations between climbing performance and the muscles’ ability to sustain submaximal anaerobic metabolic rates during all-out or submaximal, constant-intensity isometric contractions that limit blood flow or produce complete vascular occlusion. The intermittent test provides additional information on aerobic energy used for the recovery of high-energy phosphates during repetitive relaxation phases, which seems to reflect the dominant fraction of aerobic energy of this specific test modality. However, neither the latter nor any other physical or metabolic performance indicators of this specific test showed obvious links to climbing performance.

## Practical Implications

The test battery used in this study provides comprehensive assessment of both physical qualities (i.e., strength and muscle endurance) and physiological functions (i.e., local muscle aerobic and anaerobic capacity) among rock climbers. Moreover, the present test combination can be considered a new approach in functional diagnostics that can be applied in many other sports, especially in those where peripheral factors are of great importance.

The maximal force measured *via* the MVC tests, and also F_avg_ as well as FTI determined using, for example, the isometric 30-s all-out test or constant isometric contractions at 60% of F_max_, substantially explain the variance of climbing performance. Whether F_max_ from MVC test or F_avg_ from all-out tests with different durations is preferable, remains open for further analysis. F_max_ is likely the most decisive physical performance indicator in sport climbing. However, the specifics of lead climbing and bouldering with multiple subsequent contractions within 30 s and 10 min may support the idea that F_avg_ of the 30-s all-out test might provide the most relevant and economical testing approach. It is recommended that only F_avg_ can be analyzed from all-out isometric contraction tests because it appears likely that teleoanticipation will prevent achieving F_max_. Although FTI at 60% of F_max_ may be equally useful, in particular for longer lasting events such as lead climbing, it appears less economical as standardization to a given intensity requires additional testing of F_max_. However, this combined testing approach offers the possibility to directly determine anaerobic power plus anaerobic capacity. The intermittent test parameters may not correlate with climbing performance but the present results indicate that this test allows to evaluate aerobic capacity of the finger flexor muscles, which is among the abilities that should be well-developed in climbers.

## Limitation of the Study

A limitation of the study is that our inclusion criteria restricted the pool of potential participants with sufficient climbing experience required to take part in our study. The resulting small number of included climbers requires caution when generalizing the results of the study with respect to sporting level of the climbers and specific technical aspects of the testing devices used.

## Conclusion

The first analysis of performance predictors and metabolic profiles of a test battery combining the MVC test, 30-s all-out grip performance, and 60% of F_max_ continuous and intermittent grip performance until failure indicated that: (a) not only maximized grip force as performance determinant in climbing, but also, all-out isometric contractions of a given duration or the ability to sustain a given percentage of F_max_ are equally decisive physical performance indicators of climbing performance; (b) F_max_, the ability of synchronized activation of relevant motor units and thus, metabolic rate in terms of maximal anaerobic power should be tested *via* single-contraction MVC tests; (c) compared to F_max_, the F_peak_ measured during all-out tests of a 30-s duration is reduced by teleoanticipation; (d) all-out F_avg_ and FTI of constant isometric contraction at 60%F_max_ are functional measures of anaerobic capacity; (e) aerobic energy demand of the present version of an intermittent 60% F_max_ sustainability test is dominantly aerobic re-phosphorylation of high-energy phosphates during repetitive intervals, but the corresponding FTI was not decisive for climbing performance.

## Data Availability Statement

The original contributions presented in the study are included in the article/supplementary material, further inquiries can be directed to the corresponding author/s.

## Ethics Statement

The studies involving human participants were reviewed and approved by the Bioethics Committee for Clinical Research at the Regional Medical Chamber in Kraków (opinion No. 16/KBL/OIL/2013). The patients/participants provided their written informed consent to participate in this study. Written informed consent was obtained from the individual(s) for the publication of any potentially identifiable images or data included in this article.

## Author Contributions

MM and MLM designed the research, collected, analyzed, interpreted the data, drafted, edited, and revised the manuscript. MW, JS, RR, and ZS collected the data. RB interpreted the data, edited, and revised the manuscript. All authors contributed to the article and approved the submitted version.

## Conflict of Interest

The authors declare that the research was conducted in the absence of any commercial or financial relationships that could be construed as a potential conflict of interest.

## Publisher’s Note

All claims expressed in this article are solely those of the authors and do not necessarily represent those of their affiliated organizations, or those of the publisher, the editors and the reviewers. Any product that may be evaluated in this article, or claim that may be made by its manufacturer, is not guaranteed or endorsed by the publisher.
